# Band offsets of non-polar A-plane GaN/AlN and AlN/GaN heterostructures measured by X-ray photoemission spectroscopy

**DOI:** 10.1186/1556-276X-9-470

**Published:** 2014-09-04

**Authors:** Ling Sang, Qin Sheng Zhu, Shao Yan Yang, Gui Peng Liu, Hui Jie Li, Hong Yuan Wei, Chun Mei Jiao, Shu Man Liu, Zhan Guo Wang, Xiao Wei Zhou, Wei Mao, Yue Hao, Bo Shen

**Affiliations:** 1 State Key Laboratory of Artificial Microstructure and Mesoscopic Physics, School of Physics, Peking University, Beijing 100871, China; 2 Key Laboratory of Semiconductor Material Science, Beijing Key Laboratory of Low Dimensional Semiconductor Materials and Devices, Institute of Semiconductors, Chinese Academy of Sciences, Beijing 100083, China; 3 State Key Discipline Laboratory of Wide Band Gap Semiconductor Technology, Xidian University, Xi’an 710071, China

**Keywords:** GaN/AlN, Heterostructure, X-ray photoemission spectroscopy, Non-polar

## Abstract

The band offsets of non-polar A-plane GaN/AlN and AlN/GaN heterojunctions are measured by X-ray photoemission spectroscopy. A large forward-backward asymmetry is observed in the non-polar GaN/AlN and AlN/GaN heterojunctions. The valence-band offsets in the non-polar A-plane GaN/AlN and AlN/GaN heterojunctions are determined to be 1.33 ± 0.16 and 0.73 ± 0.16 eV, respectively. The large valence-band offset difference of 0.6 eV between the non-polar GaN/AlN and AlN/GaN heterojunctions is considered to be due to piezoelectric strain effect in the non-polar heterojunction overlayers.

## Background

During the last decade, group III-V nitrides are very promising semiconductor materials for application in high frequency heterojunction field-effect transistors (HFETs) [1-8]. Large band offsets at the heterojunctions are important to realize these device applications. To understand the band offsets between nitride materials at heterointerface is requested for fabricating devices. The heterojunction formed between GaN and AlN is particularly application-oriented because of their large band gap difference induced by the polarization properties of nitride materials [9-11]. Several groups have reported Δ*E*_V_ values for GaN/AlN heterojunctions fabricated by different growth techniques and determined the value of Δ*E*_V_ in a range from 0.5 to 1.4 eV [12]. However, these GaN/AlN heterojunctions reported were nearly deposited all on (0001) orientation substrates. Especially, Martin et al. reported valence-band offsets of GaN/AlN and AlN/GaN heterojunctions on C-plane sapphire substrates were 0.60 ± 0.24 and 0.57 ± 0.22 eV, respectively, both values were almost the same to each other [13]. In recent years, non-polar nitride heterostructure has drawn great interest owing to its potential applications in normally-off HEMT, high-efficiency field-free deep-ultraviolet (UV) light emitting diodes (LEDs) with wavelengths of 200 to 300 nm or sensors and so on. Non-polar nitride can eliminate internal polarization fields because of the absence of spontaneous polarization in the non-polar materials. However, the valence-band offset of non-polar A-plane GaN/AlN heterostructures has been studied by few. In this paper, we studied valence-band offsets of non-polar A-plane GaN/AlN and AlN/GaN heterostructures deposited on R-plane sapphire substrates measured by X-ray photoemission spectroscopy (XPS).

## Methods

The samples investigated were grown on R-plane sapphire substrates by metal-organic chemical vapor deposition (MOCVD). Four samples were used in our XPS experiments, namely, a 1.5-μm-thick GaN layer, 250-nm AlN layer, 5-nm GaN/250-nm AlN heterojunction, and 5-nm AlN/1.5-μm GaN heterojunction. Triethylgallium (TEGa), trimethylaluminum (TMAl), and ammonia (NH_3_) were used as the sources of Ga, Al, and N, respectively. The carrier gas was high-purity hydrogen. Before growing GaN and AlN layers, R-plane (10 $\bar{1}$ 2) sapphire substrates were thermally cleaned in H_2_ ambient at 1,000°C for 10 min to remove the adsorbed water molecules and activate sapphire surface. Then, sapphire substrates were nitridized for 5 min at 1,000°C under a mixed gas of H_2_ and NH_3_. The growth temperature of GaN and AlN layer was 1,000°C and 1,100°C, respectively. The XPS measurements were performed on a PHI Quantro SXM instrument (Physical Electronics, Inc., Chanhassen, MN, USA) with Al K_α_ radiation (*h*ν = 1,486.6 eV) at a pressure lower than 2 × 10^−9^ Torr. Charged displacement was calibrated by C 1 s (approximately 285 eV) photoelectron peak from contamination to compensate the charge effect.

## Results and discussion

**Figure 1:**
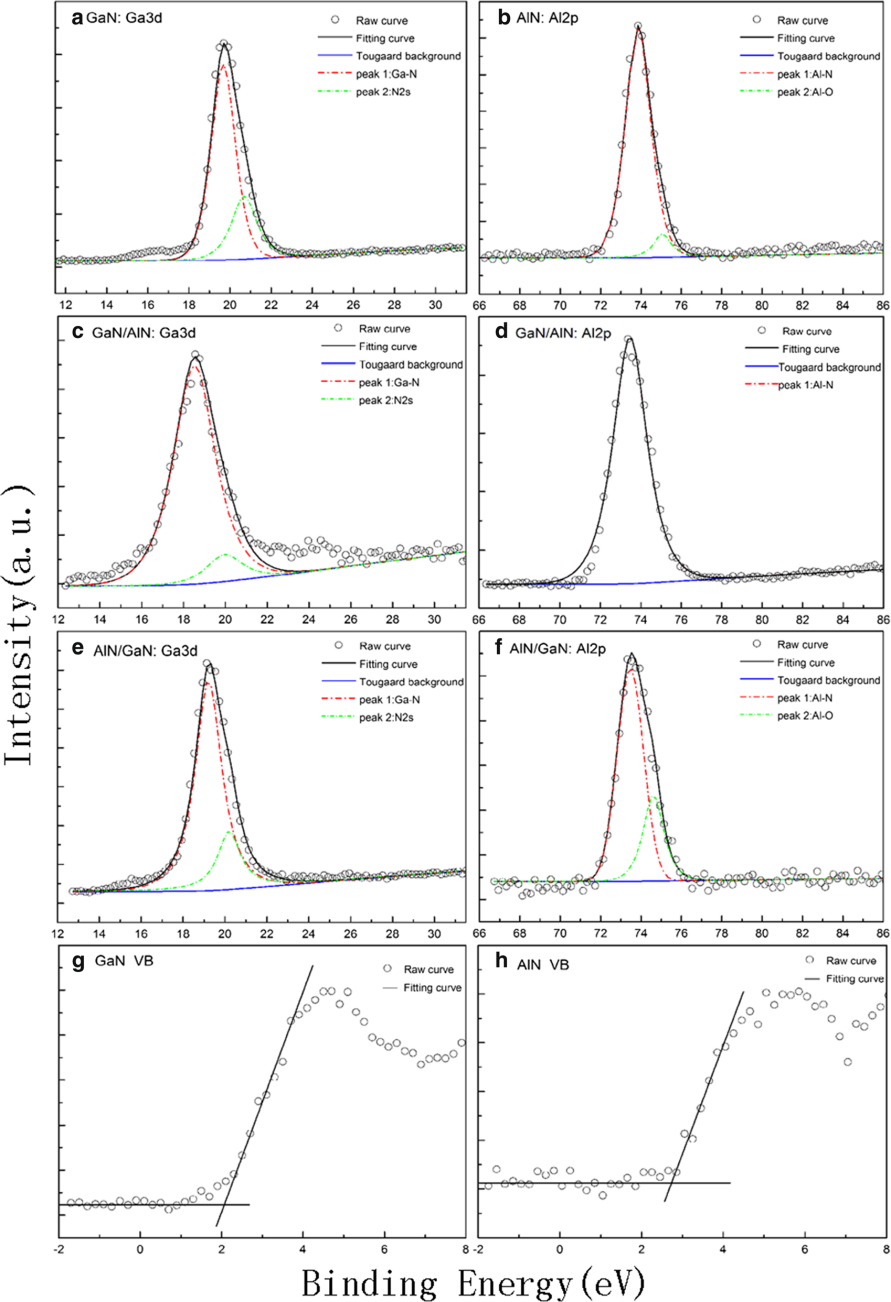
**XPS spectra of all samples.** Ga 3d XPS spectra for **(a)** GaN, **(c)** GaN/AlN, and **(e)** AlN/GaN samples and Al 2p XPS spectra for **(b)** AlN, **(d)** GaN/AlN, and **(f)** AlN/GaN. Experimental data points are fitted by Voigt (mixed Lorentzian-Gaussian) line shapes (solid lines) after the application of a Tougaard background. Also shown are VB spectra for **(g)** GaN and **(h)** AlN. The peak and VBM positions are summarized in Table 1.

The valence-band offset (*ΔE*_*V*_) can be calculated from the following formula:

(1) $$\Delta E_{V}={\left(\Delta E_{\mathrm{CL}}\right)}_{A-B}+{\left(E_{\mathrm{CL}}-E_{V}\right)}_{B}-{\left(E_{\mathrm{CL}}-E_{V}\right)}_{A}$$

where *A* and *B* represents the GaN or AlN, Δ*E*_CL_ = ±($E_{\mathrm{Ga}3d}^{\mathrm{GaN}}-E_{\mathrm{Al}2p}^{\mathrm{AlN}}$) is the energy difference between Ga 3d and Al 2p core levels (CLs), which are measured in the GaN/AlN (A-on-B) and AlN/GaN (B-on-A) heterojunction samples. (*E*_CL_ − *E*_V_) is the GaN or AlN bulk constants, which is obtained by XPS measurement on the respective thick film. Ga 3d and Al 2p XPS spectra are shown in Figure 1a,b,c,d,e,f) fitted using Tougaard backgrounds and Voigt (mixed Lorentzian-Gaussian) functions. Since considerable accordance of the fitted lines to the original measured data, the uncertainty of the Ga 3d and Al 2p core level positions were less than 0.03 and 0.01 eV, respectively, as evaluated by numerous fittings with different parameters. Figure 1a,c,e shows the Ga 3d spectrum of GaN bulk film, GaN/AlN, and AlN/GaN heterojunctions, which were fitted by two peaks, attributed to the bonding configurations Ga-N and N 2 s, respectively. For Al 2p spectrum of AlN bulk film and AlN/GaN heterojunctions shown in Figure 1b,f, two fitting peaks are attributed to the bonding configurations Al-N and Al-O, respectively. The Al-O contribution is thought to be due to oxide contamination when samples are exposed to the air. For Al 2p spectrum of GaN/AlN heterojunctions shown in Figure 1d, only one fitting peak is attributed to the bonding configuration Al-N, which coincided with the sum fitting curve shown in black solid line (red dash and dot line represents the fitting peak of bonding configuration Al-N, not shown here). The VB XPS spectra for the thick GaN and AlN samples are shown in Figure 1g,h. The VBM positions were determined by linear extrapolation of the leading edges of VB spectra to the base lines in order to account for the finite instrument resolution. The VBM positions in the GaN and AlN VB XPS spectrum are determined to be 2.06 ± 0.03 and 2.74 ± 0.09 eV, respectively. The parameters deduced from Figure 1 are summarized in Table 1 for clarity. *ΔE*_V_ in the non-polar GaN/AlN and AlN/GaN heterojunctions are calculated to be 1.33 ± 0.16 and 0.73 ± 0.16 eV, respectively, by substituting those experimental values into Equation (1).

**Table 1:** Binding energies (in eV) of the XPS peaks and VBM for GaN, AlN, GaN/AlN, and AlN/GaN samples

**Sample**	**State**	**Binding energy(eV)**	
GaN	Ga 3d	19.70 ± 0.03	Ga-N
		20.70 ± 0.03	N2s
	VBM	2.06 ± 0.03	
AlN	Al 2p	73.85 ± 0.01	Al-N
		75.05 ± 0.01	Al-O
	VBM	2.74 ± 0.09	
GaN/AlN	Al 2p	73.36 ± 0.01	Al-N
	Ga 3d	18.56 ± 0.03	Ga-N
		19.96 ± 0.03	N2s
AlN/GaN	Al 2p	73.43 ± 0.01	Al-N
		74.63 ± 0.01	Al-O
	Ga 3d	19.23 ± 0.03	Ga-N
		20.23 ± 0.03	N2s

Then, the conduction-band offsets (Δ*E*_C_) can be deduced from the following formula:

(2) $$\Delta E_{C}=\left(E_{g}^{A}-E_{g}^{B}\right)-\Delta E_{V}$$

Using room temperature band gaps for GaN and AlN (3.39 and 6.2 eV, respectively), Δ*E*_C_ in the non-polar GaN/AlN and AlN/GaN heterojunctions are calculated to be 1.48 ± 0.16 and 2.08 ± 0.16 eV, respectively, and the ratio of Δ*E*_C_:Δ*E*_V_ is close to 11:10 and 57:20, respectively.

From the calculated results, we can see that there exists a large band offset difference of 0.6 eV between the non-polar A-plane GaN/AlN and AlN/GaN heterojunctions, which may be due to the strain-induced piezoelectric fields in the non-polar films [14,15]. There is no spontaneous polarization but only piezoelectric polarization exists in the non-polar films. The heterojunction underlayers are thick enough to relax the strain caused by the lattice mismatch between GaN and AlN layers, but the heterojunction thin overlayers are only 5-nm thick and at least partially strained. The strain induces static electric fields via the piezoelectric effect. The strain-induced piezoelectric fields tend to decrease the apparent valence-band offsets for nitride materials [13].

The piezoelectric polarization *P*_PE_ via the piezoelectric effect can be given by the following [16]:

(3) $$P_{\mathrm{PE}}=e_{33}\epsilon_{\mathrm{zz}}+e_{31}\left(\epsilon_{\mathrm{xx}}+\epsilon_{\mathrm{yy}}\right)$$

Based on Hooke's law and the symmetry of wurtzite III-nitride material, the stress tensor can be represented by the following strain tensor and elastic stiffness coefficient:

(4) $$\left(\begin{array}{l} \sigma_{\mathrm{xx}}\\ \sigma_{\mathrm{yy}}\\ \sigma_{\mathrm{zz}}\\ \sigma_{\mathrm{yz}}\\ \sigma_{\mathrm{xz}}\\ \sigma_{\mathrm{xy}} \end{array}\right)=\left(\begin{array}{cccccc} C_{11} & C_{12} & C_{13} & 0 & 0 & 0\\ C_{12} & C_{11} & C_{13} & 0 & 0 & 0\\ C_{13} & C_{13} & C_{33} & 0 & 0 & 0\\ 0 & 0 & 0 & C_{44} & 0 & 0\\ 0 & 0 & 0 & 0 & C_{44} & 0\\ 0 & 0 & 0 & 0 & 0 & C_{66} \end{array}\right)\left(\begin{array}{l} \epsilon_{\mathrm{xx}}\\ \epsilon_{\mathrm{yy}}\\ \epsilon_{\mathrm{zz}}\\ \epsilon_{\mathrm{yz}}\\ \epsilon_{\mathrm{xz}}\\ \epsilon_{\mathrm{xy}} \end{array}\right)$$

where *C*_ij_ is the elastic stiffness coefficient.

For A-plane III-nitride materials, the growth plane is in the y-z plane and the in-plane strain tensor is the biaxial stress in which *ϵ*_*yy*_ = *ϵ*_*zz*_, the out-plane strain tensor is *ϵ*_*xx*_, and the other strain tensors are zero *ϵ*_*xy*_ = *ϵ*_*yz*_ = *ϵ*_*zx*_ = 0. Also, because there is not any restriction and effect along the growth direction of material, there is no stress along the growth direction, i.e., *σ*_*xx*_ = 0. Then, Equation (4) can be expressed as follows:

(5) $$\epsilon_{\mathrm{xx}}=-\frac{C_{12}\epsilon_{\mathrm{yy}}+C_{13}\epsilon_{\mathrm{zz}}}{C_{11}}=-\frac{C_{12}+C_{13}}{C_{11}}\epsilon_{\mathrm{zz}}$$

Equation (5) is substituted into the Equation (3), and we can obtain the expression of the piezoelectric polarization *P*_PE_ induced via the piezoelectric effect in the non-polar A-plane GaN/AlN and AlN/GaN heterojunctions as follows:

(6) $$P_{\mathrm{PE}}=\frac{\left(a-a_{0}\right)}{a_{0}}\left[e_{31}-\left(e_{33}+e_{31}\right)\frac{C_{11}}{C_{12}+C_{13}}\right]$$

Then, the field magnitude induced via the piezoelectric effect in the non-polar A-plane GaN/AlN and AlN/GaN heterojunctions is expressed as follows:

(7) $$E=\frac{\left(a-a_{0}\right)}{\epsilon a_{0}}\left[e_{31}-\left(e_{33}+e_{31}\right)\frac{C_{11}}{C_{12}+C_{13}}\right]$$

where *a* and *a*_0_ are the lattice constants of the materials, *ϵ* is the static dielectric constant, *e*_ij_ is the piezoelectric coefficient, and *C*_ij_ is the elastic stiffness coefficient. The values used in this work are given in Table 2[13,16-19].

**Table 2:** **Values used for calculating strain-induced piezoelectric field**[13][16][17][18][19]

	**AlN**	**GaN**
*a*_0_(Å)	3.112	3.189
*ϵ*	8.5	10.0
*e*_31_(C.m^−2^)	−0.600	−0.490
*e*_33_(C.m^−2^)	1.460	0.730
*C*_11_(GPa)	398	396
*C*_12_(GPa)	140	144
*C*_13_(GPa)	127	100

**Figure 2:**
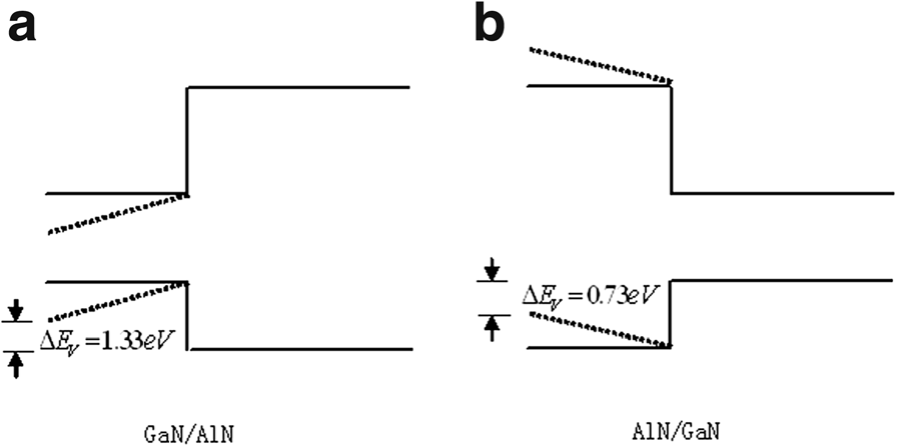
**Energy band diagram of non-polar (a) GaN/AlN and (b) AlN/GaN heterojunctions.** Apparent valence-band offsets without (the solid lines) and with (the dashed lines) a strain-induced piezoelectric field in the heterojunction overlayers.

The calculated electric fields in the strained regions are GaN(on AlN) = 2.176 × 10^8^ and AlN(on GaN) = 5.346 × 10^8^ V/m. Assuming critical heterojunction overlayer thicknesses of about 2 nm, therefore we can estimate the band offsets caused by the strain-induced piezoelectric fields in the overlayers GaN and AlN of the non-polar A-plane GaN/AlN and AlN/GaN heterojunctions to be 0.4352 and 1.0692 eV, respectively. There exists a band offset difference of 0.634 eV between them, which is almost equal to the valence-band discontinuity difference of 0.6 eV between non-polar GaN/AlN and AlN/GaN heterojunctions we measured. Figure 2 shows the energy band diagram of the non-polar A-plane GaN/AlN and AlN/GaN heterojunction. The dashed and solid lines represent the apparent energy band with and without strain-induced piezoelectric field in the heterojunction overlayers, respectively. Therefore, we can conclude that the large valence-band offsets between the non-polar A-plane GaN/AlN and AlN/GaN heterojunctions is most likely caused by the strain-induced piezoelectric fields in the heterojunction overlayers. Although we assume that the strain in the heterojunction overlayer can be released within 2 nm and obtain the above results, further analysis of the strain in the heterojunction overlayer and other physical properties will be discussed in our next paper in detail.

## Conclusions

This paper reports the study of valence-band offsets in the non-polar A-plane GaN/AlN and AlN/GaN heterojunctions evaluated by XPS technique. The valence-band offsets in the non-polar GaN/AlN and AlN/GaN heterojunctions are predicted to be 1.33 ± 0.16 and 0.73 ± 0.16 eV, respectively. There exists a large valence-band offset difference of 0.6 eV between the non-polar A-plane GaN/AlN and AlN/GaN heterojunctions, which is most likely caused by the strain-induced piezoelectric fields in the heterojunction overlayers.

## Competing interests

The authors declare that they have no competing interests.

## Authors' contributions

LS and QSZ contributed to the main ideas of AlN/GaN heterostructures design and drafted the manuscript. GPL and HJL carried out the measurement of X-ray photoemission spectroscopy. XWZ, WM, and YH carried out the MOCVD growth. SYY, HYW, CMJ, SML, ZGW, and BS gave important advices to the paper. All authors read and approved the final manuscript.
